# Supplementary material for: Application of Epidemiological Methods in a Large-Scale Cross-Sectional Study in 765 German Dairy Herds—Lessons Learned
Source: Animals (Basel). 2024 May 6;14(9):1385. doi: 10.3390/ani14091385 (PMC11083683; doi:10.3390/ani14091385)
Supplement: Supplementary file 1 [file animals-14-01385-s001.zip › S1_Overview of the areas for which data was collected_2024-01-26.pdf]

Overview of the areas for which data was collected

Description of dairies

General: farm structure, management, handling of cows

DHI-farms: data from monthly milk recordings from the last 12 months from all cows

non-DHI farms: collection of key figures from on farm documentation

HI-Tier data: data of animals on farm during the last 12 months

Future prospects of the farm

Husbandry and milking system

Layout of stalls for calves (up to 2 weeks, 3rd week of life until weaning, respectively): Type, bedding of lying areas, nesting score, hygiene, design of the barn, lighting conditions, feeding, access to water, wind protection

Layout of stalls for young animals: type of compartments, climate, lighting conditions, walking areas, lying areas, feeding areas

Layout of stalls for cows: barn compartments, climate, lighting conditions, floor, bedding areas, feeding areas, water supply, stocking density

Tie-stalls: cow comfort, type of tying, bedding, slip-resistance, dunging, measurements of cow-place

Cubicle housing: cow comfort, bedding areas, walking areas, cubicle dimensions, measurement of various barn areas

Milking system: type and equipment, personnel hygiene, milking act, cleaning and disinfection, maintenance and repair, other management

Animals

Examination of calves: ear tag, dehorning sites, auscultation of lungs, chest circumference, navel, temperature, consistency of feces, hygiene score, lameness, other diagnoses, age, sex, breed

Scoring of cows tied up: ear tag, BCS, lameness score, back line, hygiene score, hock assessment, rib swelling, skin lesions, tail fractures

Scoring of cows in cubicle housing: ear tag, BCS, hygiene score, hock assessment, neck lesions, back line, tail fracture, locomotion score

Cow size: shoulder height, oblique length of the trunk

Group observation of young animals

Fecal sample / tank milk samples for parasitological examination

Feeding

Hay production, silage production

Feeding (ration design)

Gross sensory examination of hay and silage

Feed analysis of samples of currently fed forages

Animal management

Calves: calving, stables, hygiene measures, colostrum supply, assessment of milk replacer

Pasture management

Biosecurity: structural solutions, hygiene in the animal area, purchasing and moving animals

Personality of the interviewee

Animal health, limb and udder health:

Health: around calving, measures to detect, prevent and treat diseases, vaccinations, parasite control, on farm documentation of diseases,

Diseases: observed diseases in the last 12 months, lameness observations, routine hoof care, mastitis therapy, dry-off management, udder health
