# Supplementary material for: Application of Epidemiological Methods in a Large-Scale Cross-Sectional Study in 765 German Dairy Herds—Lessons Learned
Source: Animals (Basel). 2024 May 6;14(9):1385. doi: 10.3390/ani14091385 (PMC11083683; doi:10.3390/ani14091385)
Supplement: Supplementary file 1 [file animals-14-01385-s001.zip › S2.pdf]

Descriptive evaluations operation ID_xxx

Company visit on: **01.01.2018**

Animals scored: **Cows:** 164
Calves: 34

⇨ Originally scored were: 166 cows and 34 calves
(2 cows were accidentally noted with the same ear tag or judged twice and excluded for this evaluation).

1. Evaluation cows in loose housing

Table S1: Distribution of scores skin lesions hocks, skin lesions neck, skin lesions back and tail fractures (number of animals and in percent).

	Number of cows (n) 164	%	Target values
**Ankle joints**			
No change	38	23,2	> 95%
Hairless areas	121	73,8	< 5%
Swelling	4	2,4	0%
Wound	0	0,0	0%
Wound with swelling	0	0,0	0%
Not rated	1	0,6	
**Neck**			
No change	87	53,0	
Hairless areas	70	42,7	
Wound and/or swelling	7	4,3	
Not rated	0	0,0	
**Back**			
No change	149	90,9	
Hairless areas	15	9,1	< 1%
Wound and/or swelling	0	0,0	0%
Not rated	0	0,0	
**Tail breaks**			
No tail break	105	64,0	
Tail hernia	57	34,8	
Tail amputated	2	1,2	
Not rated	0	0,0	

Table S2: Hygiene score of the udder and lower legs (number of animals and percentage)

Score	Udder (n = 164)	%	Target	lower legs (n = 164)	%	Target
Clean	106	64,6		42	25,6	
Rather clean	54	32,9		91	55,5	
Rather dirty	6	3,7	} < 5%	30	18,3	} ≤ 25%
Dirty	0	0,0		1	0,6	
n.a.	0			0		

Figure S1: Distribution of the locomotion scores within the sample (n = 164; number of animals and percentage).

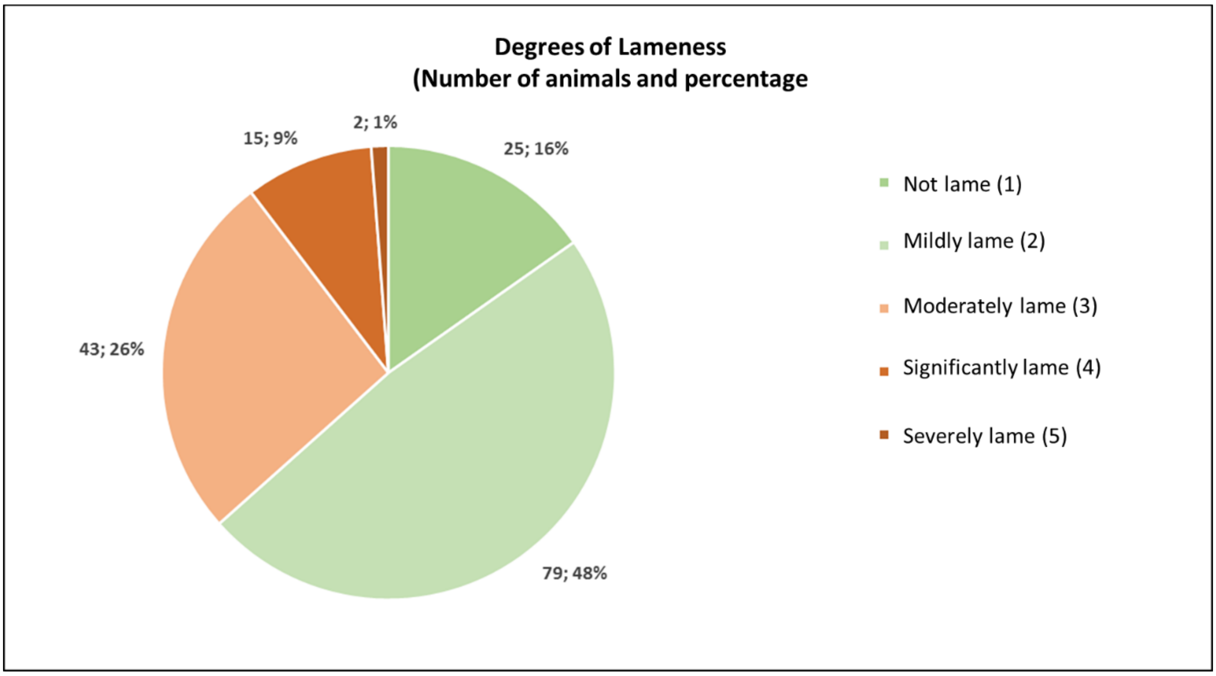

Table S3: Percentage distribution of the locomotion scores within the individual performance groups.

Power group	Animals evaluated (number n)		Lameness level				
			1	2	3	4	5
**Total**	**164**	**n**	**25**	**79**	**43**	**15**	**2**
		**%**	**15**	**48**	**26**	**9**	**1**
Group 21	36	%	11	50	28	8	3
Group 22	38	%	24	55	13	8	0
Group 23	36	%	17	47	31	6	0
Group 24	38	%	11	47	24	18	0
TSF	12	%	17	25	58	0	0
TSS	4	%	0	50	25	0	25

Figures S2 a-f: **Graphical representation of the distribution of the locomotion scores within individual performance groups with at least 4 animals (number of animals).**

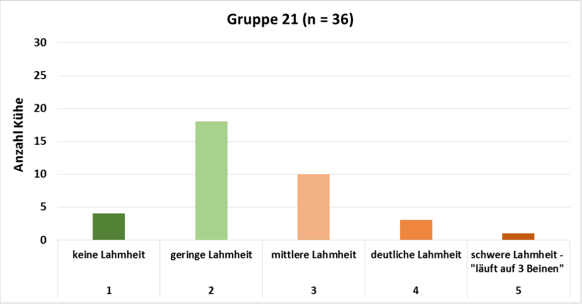

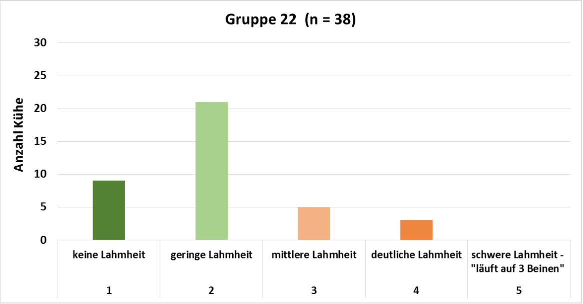

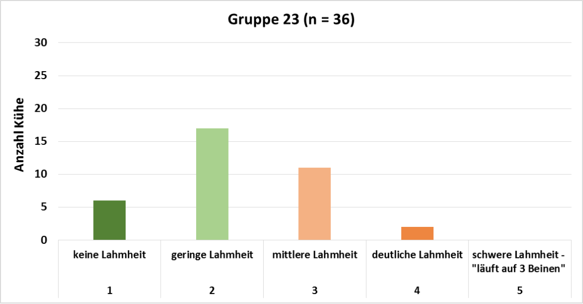

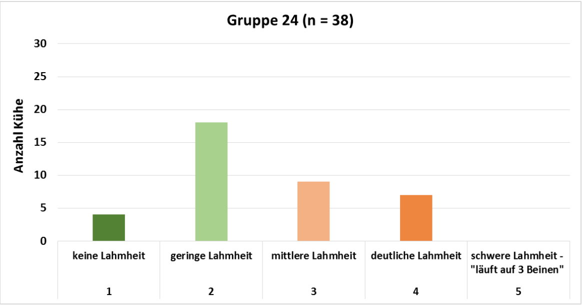

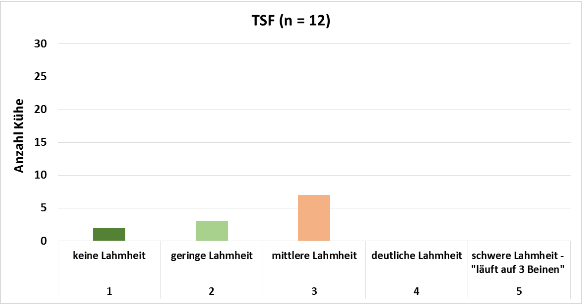

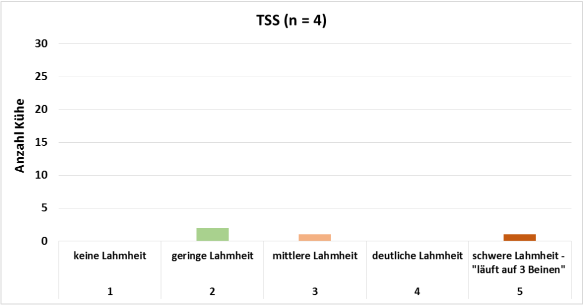

Figure S3: **Distribution of body condition scores (BCS scores) in relation to lactation day (n = 126).**

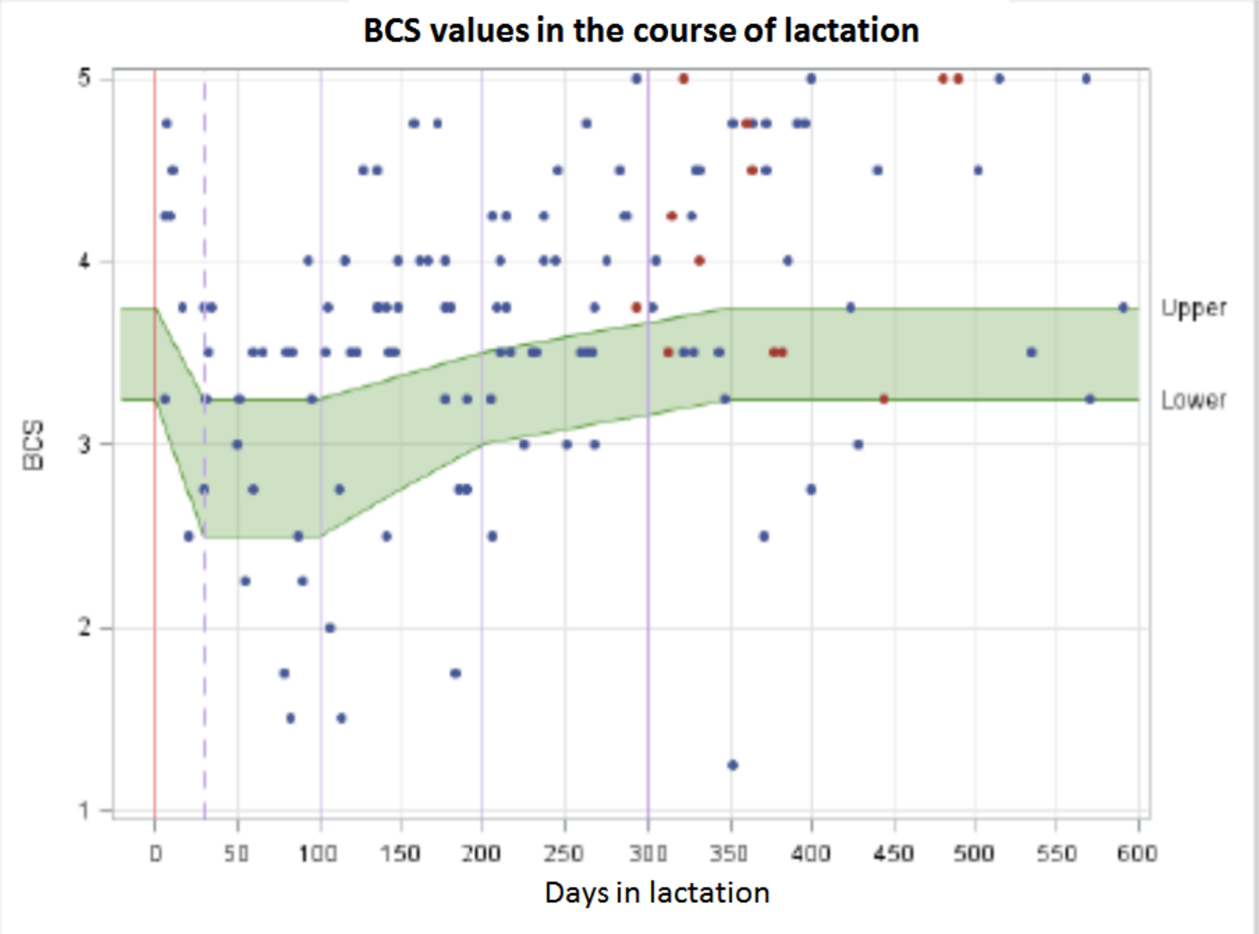

Note: The verticals (red, purple) indicate the lactation thirds assuming an average lactation duration of 300 days. Points: blue, lactating; red, dry stallion.

Table S4: **Overview of body condition distribution by lactation days (number of animals and percentage) (n = 126).**

**Performance group**	**rather thin**		**optimal**		**rather thick**	
	Number of animals	%	Number of animals	%	Number of animals	%
**Total**	**14**	**11**	**39**	**31**	**73**	**58**
0 - 30. lactation day	2	22	3	33	4	44
31 - 150th day of lactation	6	17	8	22	22	61
151 - 200 lactation day	1	8	5	38	7	54
201 - 350. lactation day	1	3	15	42	20	56
351 - 600th lactation day	4	20	4	20	12	60
Dry riser	0	0	4	33	8	67

A total of 40 cows could not be included in the evaluation for the distribution of body condition by lactation days due to one or more of the exclusion criteria listed below:

- → Ear tag numbers did not match those present on the farm
- → Cows were accidentally noted with the same ear tag number and removed for this evaluation, since a correct assignment to the lactation day was not possible
- → no indication regarding days in milk (no previous calving date)
- → Cows were other breed than SBT/RBT
- → Cows were in milk > 600 days

Table S5: Overview of the body condition of lactating and dry cows with more than 600 days post partum

Compartment	Ear tag	Body condition (score)	Days post partum
Group 00	xxxxx	rather thick (5,0)	720

2. evaluation calves

Figure S4: Overview of calf weight in relation to age

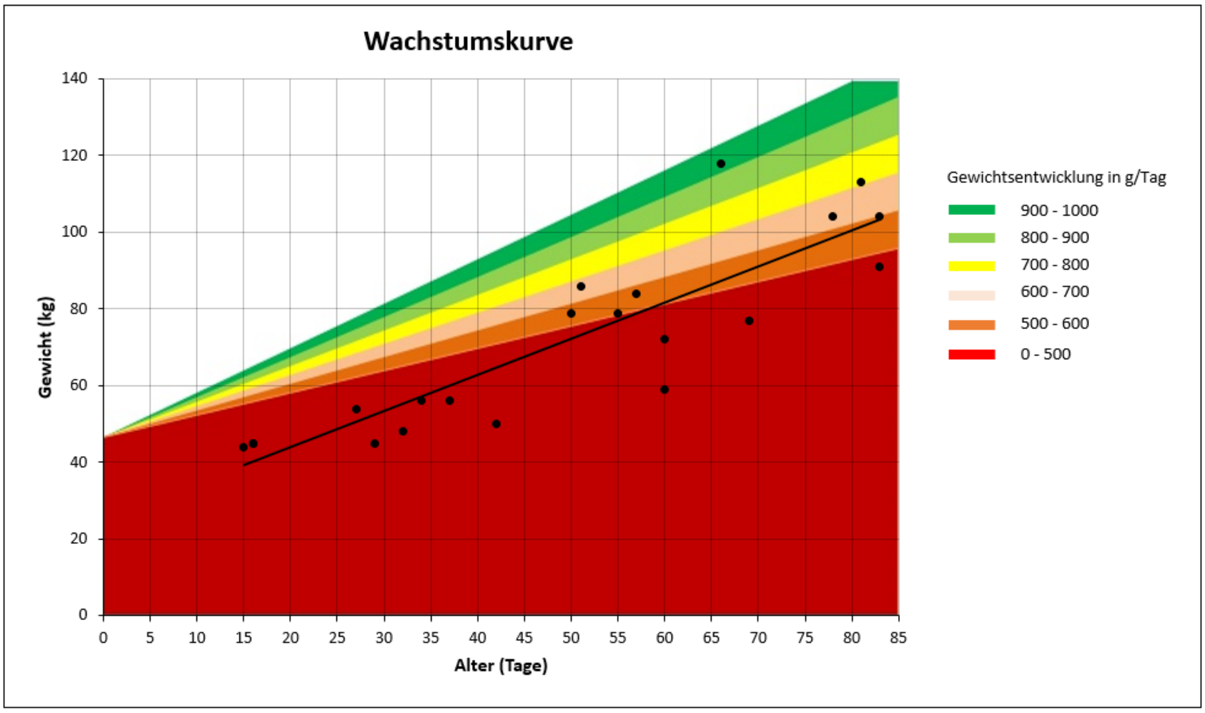

Based on data from 21 calves.

Exclusion Criteria:

→ 2 calves without 'match' (missing match with ear tags of the herd register and therefore no information regarding age).
→ 3 calves were other breed than SBT/RBT

→ 3 calves were younger than 8 days
→ 5 calves were older than 85 days

If the chest circumference is < 75 cm and the age is less than 8 days or more than 85 days, the relationship of chest circumference to body weight is not reliable.

The following calves had weight development of less than 500g per day (weight development was calculated assuming an average birth weight of 40kg): xxxx, yyyy, zzzz.

Table S6: Overview of the parameters collected from the calves and their classification (number of animals and percentage).

Parameter	Assessment levels	Number of calves	Proportion of calves (%)
**Gender**		**32**	
	female	32	94,1
	male	0	5,9
**Dehorning**		**34**	
	without any particular findings	25	73,5
	purulent secretion	5	14,7
	not dehorned	4	11,8
**Auscultation**		**34**	
Findings, general:	Breathing normally	20	58,8
	exacerbated breathing	14	41,2
	attenuated breathing	0	0,0
Findings, detailed:	Background noise	0	0,0
	Tube breathing	0	0,0
**Navel**		**34**	
	without any particular findings	27	79,4
	Inflammation	7	20,6
**Fecal consistency**		**34**	
	normal	28	82,4
	Diarrhea	5	14,7
	Not judged	1	2,9
**Hygiene**		**34**	
	rather clean	29	85,3
	Slightly dirty	5	14,7
	Highly contaminated	0	0,0
**Lameness**		**34**	
	Not lame	34	100,0
	uneven load	0	0,0

Other abnormalities on the day of the site visit:

- 2 calves had fever (ear tag 26230, 26226)
- 3 calves had cough (ear tags 26242, 26254, 20202).
- 1 calf had an umbilical hernia (ear tag 26236) and one had an umbilical abscess (ear tag 26201).
- 2 calves had bloody diarrhea (ear tag 26260, 26254).
- 2 calves showed maggot infestation after dehorning (eartag 26245, 26248).

- 1 calf exhibited circumferential growths: Neck, ventral and lateral to head left (ear tag 26216).
