# Supplementary material for: Application of Epidemiological Methods in a Large-Scale Cross-Sectional Study in 765 German Dairy Herds—Lessons Learned
Source: Animals (Basel). 2024 May 6;14(9):1385. doi: 10.3390/ani14091385 (PMC11083683; doi:10.3390/ani14091385)
Supplement: Supplementary file 1 [file animals-14-01385-s001.zip › S3.pdf]

PraeRi Benchmarking Farm XXX in region North

Number of significantly thin animals per farm (BCS <2)

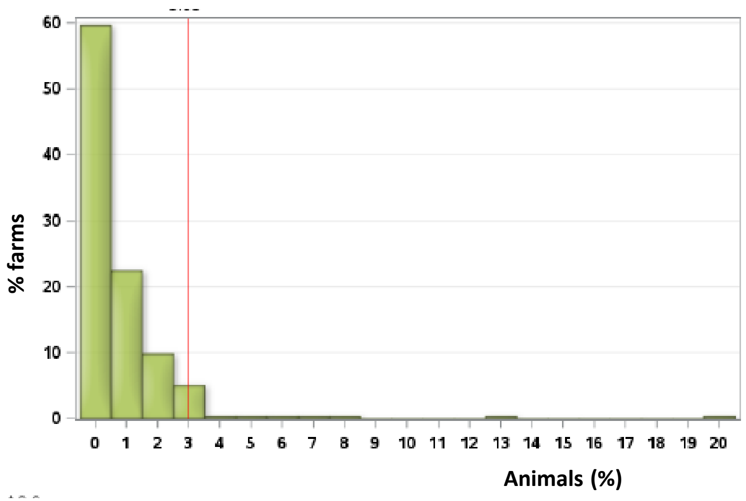

* 3.00% of scored animals in your farm were significantly thin.

Number of significantly lame animals per farm

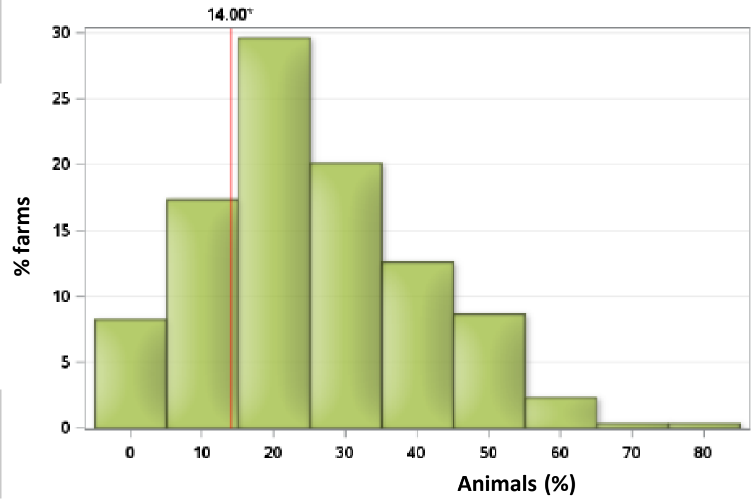

* 14.00% of scored animals in your farm were significantly lame.

Average number of per farm with critically dirty udder

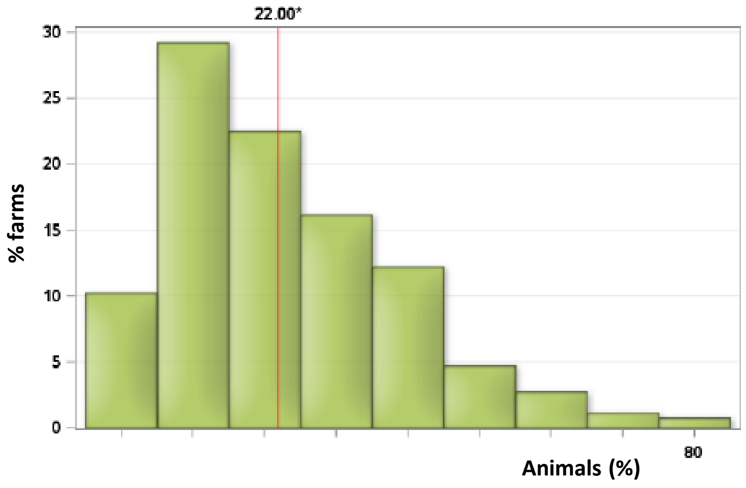

*22% of animals in your farm had a critically dirty udder

Quality of silage

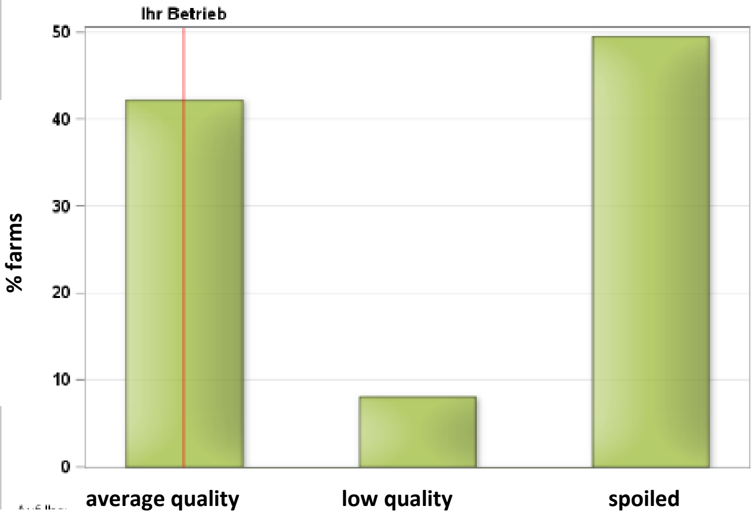

All silage samples in your farm were of average quality

Animal-feedingplace-ratio in lactating cows

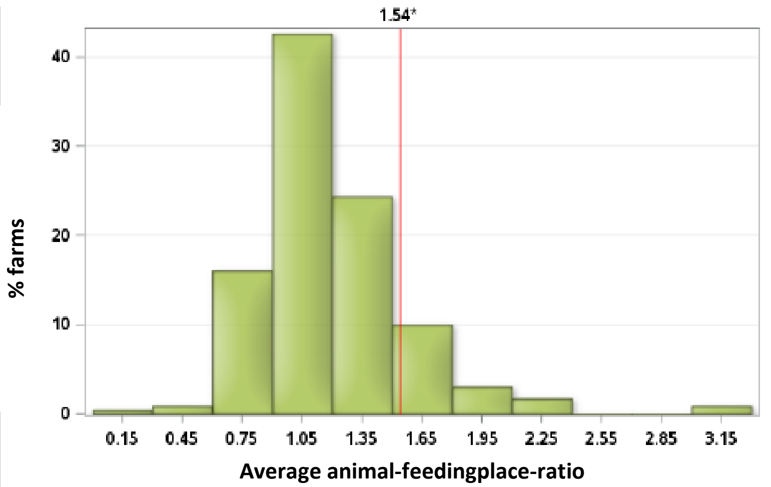

*1.54 in your farm

Animal-drinker-ratio in lactating cows

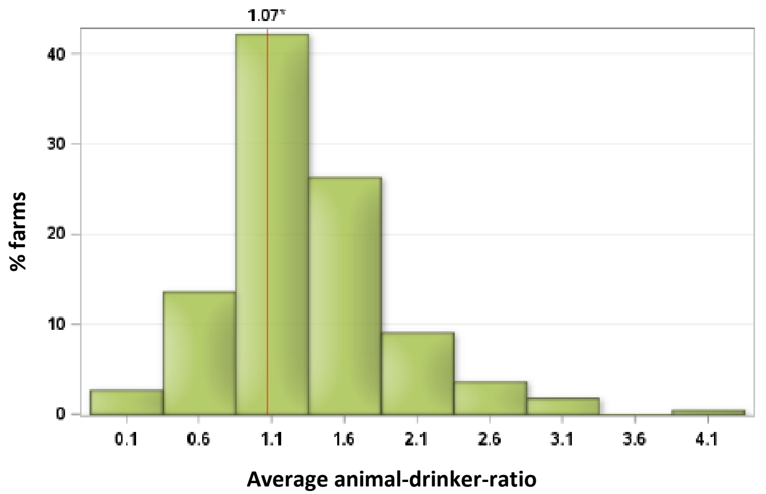

*1.07 in your farm

Mean percentage of animals with udder infection (SCC > 100,000 cells/ml milk)

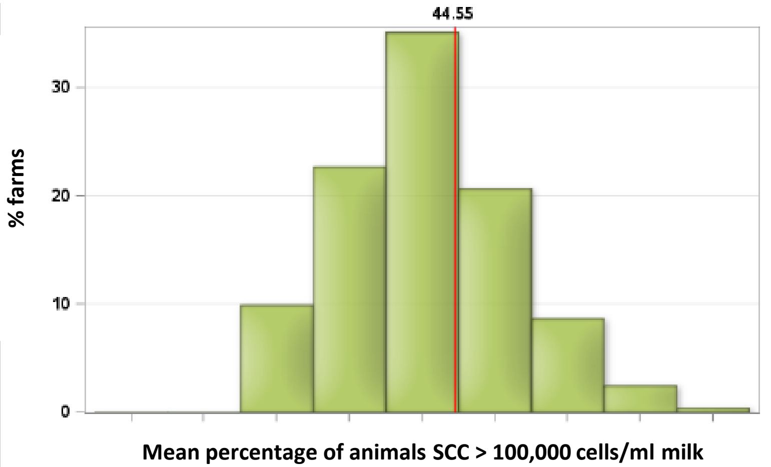

*44.55% in your farm

Percentage of dead borne calves

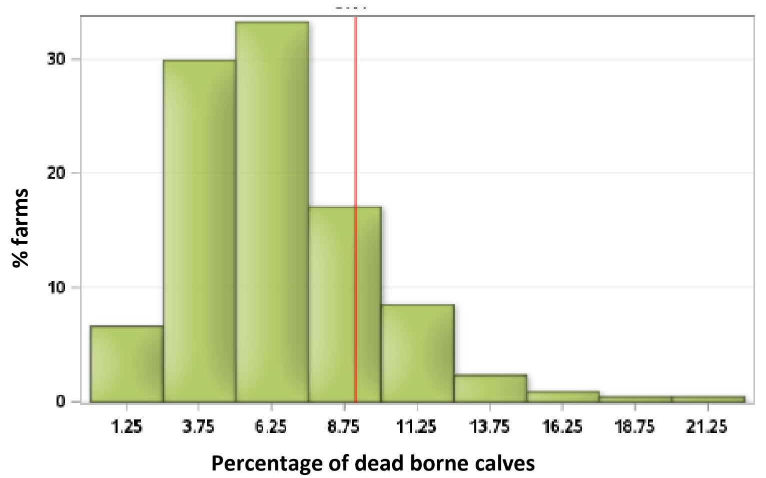

*9.14% in your farm
